# Supplementary material for: Preliminary validation of the 15-item WHO ageism experiences scale in a mixed-age UK sample
Source: PLoS One. 2026 May 5;21(5):e0347035. doi: 10.1371/journal.pone.0347035 (PMC13143091; doi:10.1371/journal.pone.0347035)
Supplement: S1 Table — (DOCX) [file pone.0347035.s001.docx]

**S1 Table. Percentage Distribution of Responses Across Ageism Items**

|  | **Response category** | | | | | |
| --- | --- | --- | --- | --- | --- | --- |
| **Item** | **1** | **2** | **3** | **4** | **5** | **NA** |
| **1** | 40.05% | 40.54% | 11.06% | 3.93% | 2.70% | 1.72% |
| **2** | 72.97% | 18.43% | 3.19% | 1.72% | 0% | 3.69% |
| **3** | 61.92% | 20.88% | 6.63% | 6.63% | 1.23% | 2.70% |
| **4** | 63.88% | 22.6% | 6.63% | 3.19% | 0.98% | 2.70% |
| **5** | 39.31% | 24.82% | 13.76% | 14.25% | 4.18% | 3.69% |
| **6** | 59.95% | 22.36% | 7.37% | 6.39% | 0% | 3.93% |
| **7** | 56.02% | 22.36% | 3.93% | 4.42% | 11.06% | 2.21% |
| **8** | 66.83% | 19.41% | 5.90% | 3.93% | 0.49% | 3.44% |
| **9** | 66.58% | 20.15% | 5.41% | 4.18% | 0% | 3.69% |
| **10** | 70.76% | 16.95% | 3.93% | 3.93% | 0.98% | 3.44% |
| **11** | 75.43% | 13.51% | 3.44% | 2.46% | 0.74% | 4.42% |
| **12** | 63.39% | 20.88% | 6.63% | 4.42% | 0.49% | 4.18% |
| **13** | 24.32% | 13.76% | 19.16% | 23.34% | 12.53% | 6.88% |
| **14** | 6.39% | 22.60% | 36.12% | 19.90% | 10.57% | 4.42% |
| **15** | 53.81% | 16.71% | 8.11% | 7.13% | 1.72% | 12.53% |

*Note*. Values represent the percentage of participants selecting each response category. Response options range from 1 (lowest ageism) to 5 (highest ageism). Items were reverse-coded prior to tabulation for consistent interpretation.
