# Supplementary material for: Preliminary validation of the 15-item WHO ageism experiences scale in a mixed-age UK sample
Source: PLoS One. 2026 May 5;21(5):e0347035. doi: 10.1371/journal.pone.0347035 (PMC13143091; doi:10.1371/journal.pone.0347035)
Supplement: S3 Table — (DOCX) [file pone.0347035.s003.docx]

**S3 Table. Factor Loadings for Self-directed Ageism Two-factor Solution**

|  | **Factor 1** | **Factor 2** |
| --- | --- | --- |
| At my age, my life has plenty of purpose | -0.22 | -0.29 |
| I am a burden because of my age | 0.01 | 0.96 |
| I am embarrassed of my age | 0.47 | 0.20 |
| Due to my age, I limit my participation in discussions even when they are about things that affect me | 0.62 | 0.18 |
| There are things I would like to do if I did not consider them inappropriate for my age group | 0.69 | -0.13 |
