# Supplementary material for: Preliminary validation of the 15-item WHO ageism experiences scale in a mixed-age UK sample
Source: PLoS One. 2026 May 5;21(5):e0347035. doi: 10.1371/journal.pone.0347035 (PMC13143091; doi:10.1371/journal.pone.0347035)
Supplement: Attachment — Submitted filename: Response to reviewer_who ageism 15_plosone_FINAL.docx [file pone.0347035.s005.docx]

**PONE-D-25-50563**

**Response to Reviewers**

Dear Dr. Antony Bayer,

Thanks for giving us the opportunity to submit a revised draft of the manuscript “Preliminary validation of the 15-item WHO ageism experiences scale in a mixed-age UK sample” for publication in *PLOS One*. We appreciate the time and effort that you and the reviewers dedicated to providing feedback on our manuscript and are grateful for the insightful comments on and valuable improvements to our paper.

We have incorporated suggestions made by the reviewers. Those changes are highlighted within the manuscript. Please see below, in blue, for a point-by-point response to the reviewers’ comments and concerns, and quoted text from the manuscripts is in purple.

**Reviewers' Comments to the Authors:**

**Reviewer #1:**

This paper presents a preliminary validation of the 15-item WHO ageism experiences scale in a mixed-age UK sample, aiming to address a documented gap in the availability of psychometrically sound measures of ageism. The study's focus on capturing the multidimensional nature of ageism across different age groups and contexts is potentially valuable, considering limitations in the existing measurement landscape.

**Response**: We thank the reviewer for the careful reading of the manuscript and for the positive assessment of the study's goal. We believe that the development of a valid and reliable measure capable of capturing the multidimensional nature of ageism, as called for by the WHO, is critical to advancing global efforts to combat this issue. We hope this preliminary validation study serves as an important first step in introducing the 15-item WHO ageism experiences scale to a wider audience and providing the necessary initial psychometric evidence to foster confidence in its adoption for future research.

Strengths:

• Relevance and Timeliness: The research aligns with the WHO's global campaign to combat ageism and responds to the urgent need for reliable tools to measure ageism experiences.

• Clear Conceptual Framework: The paper defines ageism and its dimensions (self-directed, interpersonal, institutional) providing a rationale for the scale's development.

• Sound Methodology: The use of exploratory factor analysis, internal consistency, test-retest reliability, and convergent validity offers an initial evaluation of the scale's properties.

• Promising Initial Findings: The results suggest initial good internal consistency and test-retest reliability.

**Response**: We appreciate the reviewer highlighting the relevance and timeliness of this research, particularly its alignment with the WHO mandate to combat ageism, and its clear conceptual framework. We are encouraged that the reviewer acknowledges the sound methodology employed and agrees that the promising initial findings support the scale's internal consistency, test-retest reliability, and potential for broader use.

Areas for Improvement (Critical Concerns):

**Comments1-1:** Sample Size and Selection: The justification for the sample size (N=402) relies on resource constraints. While this is understandable, there's a lack of clear explanation concerning the adequacy of the sample size. In addition, elaboration on the selection criteria of the sample is needed. What are the specific inclusion/exclusion criteria?

**Response**: We thank the reviewer for raising these critical points regarding sample size and participant selection.

1. **Sample size and adequacy:** We acknowledge that our sample size was influenced by resource constraints, and we fully agree that a larger sample would have been preferable. However, the achieved sample size of n = 402 meets and exceeds several widely cited recommendations for factor analysis and scale validation. Reviews on sample size requirements for psychometric validation (Anthoine et al., 2014; Gunawan et al., 2021) suggested that although guidelines vary across studies, there is broad agreement that sample sizes of 50 are considered “very poor,” 100 “poor,” 200 “fair,” 300 “good,” 500 “very good,” and ≥1,000 “excellent”. Moreover, a complementary rule-of-thumb is the item-to-response ratio (*p*:N), with recommended ratios ranging between 1:3 and 1:20. Given that our scale contains 15 items and our sample includes 402 participants, our ratio is approximately 1:26.8, which is above the recommended ranges. To clarify this in the manuscript, we have added the following text: “The sample size requirements depend on properties of the items such as factor loadings that cannot be known in advance, therefore, sample size planning for scale validation can be challenging. The sample size for the present study was based on a resource constraint approach in which the number of participants was maximised within the available resources whilst ensuring that it would meet minimal sample size requirements for psychometric validation for scales of its length. For example, Rouquette and Falissard (2011) conducted a simulation study, recommending a minimum sample size of 300 for the effective validation of 15-item scales. This size is also considered 'good' according to other established psychometric guidelines (Anthoine et al., 2014; Gunawan et al., 2021). Therefore, our sample size aligned with those criteria, allowing us to perform thorough psychometric evaluations.”
2. **Selection criteria:** We agree with the need for greater clarity on inclusion/exclusion criteria. We clarify that participants were recruited via the Prolific platform using convenience sampling. Inclusion criteria were: being an adult (age 18+), residing in the UK, and being able to provide informed consent. Exclusion criteria were: identifying as a student, and being unable to provide informed consent. We also aimed to achieve a gender-balanced sample, which resulted in 198 female and 199 male participants being included. We have updated the main text to reflect these specific criteria more explicitly. The revised text reads as follows on Method-participants section. “Inclusion criteria required participants to be adults (age 18+), able to provide informed consent, and based in the UK. Exclusion criteria included identifying as a student or being unable to provide informed consent.”

**Comments1-2:** Item Selection and Justification: The paper starts with 15 items and concludes with 15 items. The stability raises concern about content validity. The selection criteria for the scale's 15 items from the WHO ageism item pool are inadequately justified. The rationale for why these specific 15 items were chosen to cover all relevant aspects of ageism needs to be transparent. It is not clear if it sufficiently captured key aspects of the construct of interest, raising doubts about content validity.

**Response:** We appreciate the reviewer's concern regarding the transparency of the 15-item selection process and its relationship to content validity. We clarify that the final 15-item scale was selected prior to this validation study from a comprehensive item pool developed by Murray and de la Fuente-Núñez (2023). The selection process was driven by a commitment to maximising content validity, prioritising both relevance and representativeness of the core ageism domains. Following the framework of(Haynes et al., 1995), we defined content validity as ‘the degree to which the elements of an assessment are relevant to and representative of the targeted construct for a particular assessment purpose’. Item selection was, therefore, driven by expert judgement regarding how well specific items satisfied these two criteria relative to others in the comprehensive item pool. Specifically:

- Cover all key domains align with Levy (2009)’s definition and the Global report on Ageism from WHO. Specifically, it spanned three components (stereotypes, prejudices, and discrimination), at three levels (self-directed interpersonal, and institutional level). To our knowledge, this is the first scale that can adequately capture all three aspects with respect to ageism experiences.
- Prioritise general items to capture overall exposure across contexts, making the scale feasible for use across diverse ages groups and countries.
- Balance the scale, including a mixture of positively and negatively worded items to manage the risk of an overly negative scale (which happens in many other scales about age discrimination, or age stereotypes)
- Capture a rage of severity range, from *everyday* to more *extreme*.
- Give proportionally greater representation to discrimination items to reflect the assumption that the behaviour aspect is the most direct and impactful form of ageism.

To further increase transparency regarding the selection process and content validity considerations, we have added a discussion of the specific criteria (relevance and representativeness) to the Method section of the manuscript. Additionally, we have included a brief discussion of items that were not selected (e.g., 'Others feel anger towards me because of my age' was excluded as it was deemed conceptually redundant or less optimal than a similar, less extreme item) and have indicated that the full non-selected item pool is available in the supplemental materials of the item development paper (Murray & De La Fuente-Núñez, 2023).

The following texts were added to the main texts: “The item selection process was primarily driven by expert judgement aimed at satisfying the criteria for content validity. Content validity was conceptualised as the extent to which the components of the measure appropriately and thoroughly reflect the target construct for a given assessment goal. Specifically, two key features of content validity are relevance and representativeness (Haynes et al., 1995). The former refers to the appropriateness of an aspect of a scale to the target construct and the latter to the extent to which the facets of the target construct are proportionally represented by items. Thus, for a scale to possess content validity, it should include items covering the full range of possible manifestations both in terms of specific types of symptoms and in terms of ‘severity’ in proportion to the target construct in the population.”

**Comments1-3:** Construct Validity Concerns: The absence of a measure of self-directed ageism for construct validation is an oversight. Including such a measure would have provided a stronger test of whether the scale is indeed measuring ageism experiences.

**Response:** We thank the reviewer for this insightful comment regarding the construct validity of the self-directed ageism component. We acknowledge that the absence of an external measure specifically assessing self-directed or internalised ageism is a limitation of the present study and restricts our ability to fully establish the convergent validity of this subscale. The Perceived Ageism Questionnaire (PAQ) was selected because it captures both stereotypes and discrimination, and includes both negative and positive ageism, making it a useful comparator for the interpersonal and institutional dimensions. However, as highlighted in a recent review of ageism experience measures (Murray & Li, 2025), no existing instrument, except the WHO Ageism Experiences Scale, adequately covers all levels and dimensions of ageism experiences.

We have now added this point to the limitations section of the manuscript and suggested that future research should incorporate an external measure specifically targeting self-directed ageism to more rigorously evaluate the convergent validity of this subscale. In Limitation section, we added: “Moreover, another limitation of the current validation study is the absence of an external measure specifically designed to capture internalised or self-directed ageism for convergent validation. While the Perceived Ageism Questionnaire (PAQ) was included for its ability to measure both stereotypes and discrimination, and its coverage of both negative and positive ageism, it does not contain items targeting the self-directed dimension. This restricts the ability to fully establish the convergent validity of the self-directed ageism subscale”. We also added “Future research should incorporate an established external measure of internalised ageism to more rigorously evaluate the convergent validity of this specific subscale. One suitable option is the Aging Perceptions Questionnaire (APQ; Barker et al., 2007), which assesses individuals’ beliefs and feelings about their own ageing and has been widely used as a measure of self-directed or internalised ageism. The APQ also offers shorter validated versions (e.g., the Brief APQ [B-APQ] and the Short APQ [APQ-S]), making it feasible to include in future studies alongside the current scale.”

Despite this limitation, several indicators support the internal validity of the self-directed ageism subscale. The exploratory factor analysis yielded a clear one-factor solution for the self-directed items, suggesting that they coherently represent a single underlying construct. In addition, the subscale demonstrated good internal consistency and strong test-retest reliability, providing further evidence that the items reliably measure the intended construct.

**Comments1-4:** Deeper Dive into Construct Validity: While convergent validity is assessed, further investigation into discriminant validity would strengthen the argument that the scale measures ageism specifically, and not other related constructs.

**Response:** We thank the reviewer for this comment. We agree with the reviewer that further investigation into discriminant validity would significantly strengthen the argument for the scale's construct validity, demonstrating that it measures ageism experiences distinct from other psychological constructs. While the current study focused on convergent validity using the PAQ, and provided some initial interpretive illumination by assessing associations with sociodemographics, we did not include a measure of a theoretically distinct construct (e.g., general negative affect) to rigorously test discriminant validity. We recognise this as an important area for improvement. We have added a statement to the Discussion section, under Strengths and Limitations, to underscore the need for future studies to incorporate measures that allow for a robust assessment of discriminant validity. The added text reads as follows: “Finally, there were several psychometric properties which we did not test in the current study due to data limitations, which could be assessed in the future, including correlations with a wider range of criterion variables, investigation of discriminant validity with measures of distinct constructs (e.g., general negative affect), measurement invariance across core groups (e.g., age, gender, and health status); and sensitivity to interventions (Mokkink et al., 2010).”

**Comments1-5:** Exploring Subgroup Differences: The authors acknowledge the lack of age-stratified analyses. However, exploring potential differences in ageism experiences based on other sociodemographic variables (e.g., gender, ethnicity) could provide valuable insights into the scale's performance across diverse subgroups.

**Response:** We thank the reviewer for highlighting the importance of exploring potential differences in ageism experiences across various sociodemographic subgroups.

We acknowledge the limitations of our preliminary validation study: 1) While we employed a mixed-age UK adult sample, we recognise the sample was largely ethnically homogeneous (primarily White British). This restricts the immediate generalisability of the findings across diverse ethnic groups within the UK and globally; 2) Due to insufficient numbers in specific age bands, we could not conduct age-stratified analyses. However, we have included initial analyses on subgroup differences, as presented in Table 4, which showed that the association between age (and age^2^), sex, and health status, with both the self-directed and interpersonal/institutional domains.

We agree that exploring additional variables (such as ethnicity) and conducting robust testing for measurement invariance is crucial for future work to ensure the scale performs consistently across diverse subgroups. We have updated the Limitations section to explicitly address the ethnic homogeneity and reinforce the need for future testing across age, gender, ethnicity, and health status. The added texts read as follows: “It is important to note the limitations of the present study. First, our sample was a non-probability sample and is not representative of the wider UK population. Crucially, while the sample was drawn from the UK, it was largely ethnically homogeneous (primarily White British), which restricts the generalisability of the findings across diverse ethnic subgroups.” “However, further work will be required to extend the validation to other populations; including the adaptation and translation of the scale for use in a diversity of additional global contexts and ethnically diverse groups”. “Finally, there were several psychometric properties which we did not test in the current study due to data limitations, which could be assessed in the future, including correlations with a wider range of criterion variables, investigation of discriminant validity with measures of distinct constructs (e.g., general negative affect), measurement invariance across core groups (e.g., age, gender, ethnicity, and health status); and sensitivity to interventions (Mokkink et al., 2010).”

**Comments1-6:** Clarity on Formative vs. Reflective: The paper's discussion of formative vs. reflective indicators requires further clarification. The statement "For formative/causal indicators, psychometric measurement models such as a factor analysis and item response theory, which assume an underlying latent trait are not appropriate" needs to be explained more thoroughly. A more accessible explanation of the underlying statistical assumptions and why they are violated in the case of formative indicators is necessary.

**Response:** We thank the reviewer for highlighting the need for greater clarity and accessibility in the discussion of formative versus reflective indicators. As this distinction is foundational to the psychometric approach taken in the present study, specifically for the self-directed items (reflective) versus the interpersonal/institutional items (formative), we agree that a more thorough explanation of the underlying statistical assumptions is needed.

For self-directed ageism, the corresponding items (e.g., “I am embarrassed of my age” or “I am a burden because of my age”) are treated as reflective indicators. They are viewed as the manifestations or effects of an internal, latent psychological trait. Conceptually, if an individual holds a high level of this internal trait, they are likely to report multiple, consistent negative self-directed thoughts, feelings, and behaviours. Since these item responses all stem from the same hypothesised internal cause, these indicators are expected to strongly correlate and are appropriately tested using models that assume a latent trait, like EFA or CFA.

In contrast, Interpersonal and Institutional ageism items (e.g., “Others make decisions for me because of my age” or “Policies made by the government... do not meet the needs of people my age”) are treated as formative indicators. They represent a composite of external ageist events/environments experienced by the individual across various domains. A high score on this subscale does not mean a person possesses a single, unitary internal trait; rather, it means they have been exposed to a variety of distinct, heterogeneous external ageism experiences. Because these external causes are heterogeneous and *define/form* the construct rather than *reflect* a latent trait, they are not assumed to be highly correlated. This distinction is important because it necessitates the use of different validation methods, focusing on test-retest reliability and convergent validity rather than internal consistency and factor analysis for the formative components.

We have revised the Statistical Procedure subsection to provide a clearer explanation of why traditional psychometric models, particularly those based on factor analysis, are inappropriate for formative indicators. The text we added reads as follows:

“Murray & De La Fuente-Nunez (2023) argued that some of the ageism experiences items in the WHO-ageism item pool, i.e., the interpersonal and institutional items have formative (or ‘causal’) rather than reflective (or ‘effect’) relations with the concepts they measure (Murray & Booth, 2018). This is based on the idea that for interpersonal and institutional ageism indicators a person’s overall experiences of ageism do not reflect an internal latent (psychological) trait. Rather, they are better conceptualised as a composite of experiences across different contexts, and in interaction with multiple different individuals and institutions. For example, a person’s experience of interpersonal prejudice (e.g., “Other people feel uncomfortable around me because of my age”) and their experience of institutional discrimination (e.g., “Policies made by the government do not meet the needs of people my age”) are distinct external experiences that form the overall ageism score, and these experiences are not assumed to be necessarily highly correlate. Self-directed ageism, in contrast can be thought of as reflecting a psychological trait of internalised ageism. The items in this domain are considered reflective indicators, meaning they are the effects or manifestations of this single underlying internal trait.”

“For formative/causal indicators, psychometric measurement models such a factor analysis and item response theory, which assume an underlying latent trait are not appropriate (Bollen & Bauldry, 2011; MacKenzie et al., 2005). This is because these models are built on the statistical assumption that all indicators are measuring the same latent cause. When indicators *define* the construct rather than *reflect* it (as in the formative model), this fundamental assumption is violated, making tests like internal consistency reliability and factorial validity conceptually unsound for assessing their psychometric quality. However, formative indicators could still be expected to show high test-rest reliability and convergent and criterion validity with other measures. As such, our analyses of the interpersonal and institutional experiences items focus on these properties. Our analyses of self-directed ageism additionally include analyses of the internal structure and internal consistency reliability of the items.”

**Comments1-7:** Factor Analysis Limitations: The authors did not attempt to conduct Factor Analysis on interpersonal and institutional ageism. The explanation for why it’s not possible remains somewhat unclear. If there is a good reason, it has to be clearly explained so that the readers can fully understand the decision.

**Response:** We acknowledge the reviewer’s request for a clearer explanation of why Factor Analysis was not attempted for the interpersonal and institutional ageism items. Our decision is based on the conceptualisation of these items as formative (or causal) indicators, a rationale consistent with the scale's development, the WHO user manual, and established psychometric literature. We have already revised the Statistical procedure: Overview section to provide a more detailed and accessible explanation of this distinction, clarifying that for these formative components, we appropriately relied on test-retest reliability and convergent validity for psychometric support. Please see our answers for Comments 1-6 for more details.

**Comments1-8:** Dimensionality of Ageism: The authors should have explained why they didn’t attempt to conduct FA to see whether the 3 dimensions of ageism can be teased apart- e.g., intra, inter, and institutional ageism. Addressing the scale's ability to differentiate these dimensions is essential for its usefulness in understanding and addressing ageism.

**Response:** We thank the reviewer for requesting clarification on why FA was not performed on the overall scale’s three levels of ageism manifestation.

The decision is based on the scale's conceptual framework. Self-directed ageism is treated as a psychological trait (internalised ageism), making it appropriate for FA. We did conduct FA on these items, and the results supported a single underlying factor. The interpersonal and institutional ageism components are different. They represent a composite of external experiences but not a single latent internal trait. Because FA relies on the assumption of a single underlying trait, applying it to these heterogeneous experiences would be methodologically unsound. For these experience-based domains, we appropriately relied on test-retest reliability and convergent validity for psychometric evidence.

**Comments1-9:** Floor and Ceiling Effects: The paper does not address potential floor or ceiling effects. Given that the study indicates "overall low levels of ageism," it is critical to examine whether the scale is sensitive enough to detect ageism experiences across the entire spectrum.

**Response:** We thank the reviewer for identifying the need to assess potential floor and ceiling effects, particularly given the finding of overall low levels of ageism in our sample.

We have now conducted additional analyses to address this. We calculated the percentage of participants who selected each response option (1, 2, 3, 4, 5) and the rate of missing data (NA) for all 15 items. This detailed breakdown has been included as a new Supplementary Table S1.

Descriptive analysis of the data confirms a clustering of responses towards the low end for the majority of items, while we think that this distribution is within our expectation and does not reflect a critical limitation of the scale's sensitivity, but rather due to the nature of the current sample and alignment with existing literature. Specifically, our sample is a mixed-age adult UK sample (ages 20-65), which is not considered a high-risk group for pervasive ageism experiences. The low means and high frequency of the lowest response option accurately reflect the low prevalence of explicit ageism experiences in this population. In addition, this response pattern is actually common for age discrimination measures in population-based surveys. Prior literature (Jackson et al., 2019; Rippon et al., 2015) on age discrimination in cohorts like the English Longitudinal Study of Ageing (ELSA) and the Health and Retirement Study (HRS) has shown that responses are inherently highly skewed towards the *never experienced* category, even in older-adult cohorts. To manage this, these studies often resorted to dichotomising responses, where the lowest category (e.g., never experienced) was grouped against all other responses (had experience). Therefore, we conclude that this distribution is a true reflection of the low prevalence of ageism experiences in a general, mixed-age sample, and thus does not indicate a fundamental issue with the scale.

We have addressed the reviewer’s concern regarding floor effects by including the detailed item-level response distributions (Supplementary Table S1) in the manuscript (and Result Section). Furthermore, we have added a discussion in the Strengths and Limitations sections to contextualise the findings, ensuring that readers are fully informed about the scale’s performance in this context and understand the methodological rationale.

Specifically, in Result section, we added “The full breakdown of response frequencies (percentages for scores 1 to 5 and missing data) for each item is provided in Supplementary Table S1, which showed a pattern of responses reflecting low agreement with perceived ageism experiences.”

In Discussion (general) section, we added “Finally, while detailed analysis of item response distributions suggested that most participants selected the response category indicating lowest ageism level, this pattern is highly consistent with our expectations given the nature of the current sample and alignment with existing literature. Specifically, the sample is a mixed-age sample, which is not considered a high-risk group for ageism experiences, and the observed low means accurately reflect the low prevalence of explicit ageism experiences in this population. Prior literature on age discrimination measures in large-scale, population-based surveys like ELSA and HRS has consistently shown that responses are highly skewed towards the “never experienced” category, even in older-adult cohorts. Thus, we concluded that the observed distribution is a representation of the phenomenon in this population.”

And in Discussion (strength and limitation) section, we added “Future validation studies in populations where ageism is expected to be more prevalent (e.g., older adult clinical samples or specific cultural setting) will be essential to test the scale’s full range sensitivity assess the extent of floor effect.”

**Comments1-10:** Discriminant Validity: The omission of an assessment of discriminant validity is a significant weakness. Demonstrating that the scale measures something distinct from related constructs (e.g., general negativity, depression) is essential for establishing its unique contribution.

**Response:** We thank the reviewer for this important comment. We agree with the reviewer that the omission of an assessment of discriminant validity is a significant weakness in this preliminary validation study. We recognise this as a key area for expansion in future work. We have updated the Limitations section to explicitly acknowledge this gap and the need for future studies to incorporate measures that allow for a robust assessment of discriminant validity. The revised texts were as follows: “Finally, there were several psychometric properties which we did not test in the current study due to data limitations, which could be assessed in the future, including correlations with a wider range of criterion variables, investigation of discriminant validity with measures of distinct constructs (e.g., general negative affect), measurement invariance across core groups (e.g., age, gender, ethnicity, and health status); and sensitivity to interventions (Mokkink et al., 2010).”

**Comments1-11:** Justification for Formative Interpretation: "However, a formative interpretation is arguably more suitable for the interpersonal and institutional ageism items as ageism experiences from the external environment can combine from heterogeneous sources, namely, a diversity of individuals and institutions, rather than a single unitary factor”. There are no clear reasons or more detail description to fully support the claim.

**Response:** We thank the reviewer for requesting a more detailed justification for the core methodological decision to adopt a formative interpretation for the interpersonal and institutional ageism items in the Discussion section. We agree that this distinction is crucial and requires robust conceptual support. We then clarified the distinction between the two types of measurement models.

1. Reflective indicators (effect indicators), which can be viewed as manifestations or effects of internal, latent psychological trait. For example, the symptoms of depression (e.g., persistent sadness, loss of interest, fatigue) are reflective indicators, as they are all should be caused by the same underlying psychological disorder (the latent trait of depression). Since all indicators (symptoms) share a single common cause (depressi0n), they are expected to be dependent and correlated with one another. This model is suitable for testing through factor analysis internal consistency reliability, since they require a single underlying latent trait.
2. Formative Indicators (causal indicators), which can be viewed as components that combine or form the overall construct. The construct is defined by the indicators, which are seen as external causes. One sample is Socioeconomic Status (SES) which is formed by indicators like income, education, and occupational prestige. These indicators are external causes/determinants of SES, and to be high SES, one should have high income, should have high education, or should have high occupational prestige. Those indictors can have +, -, or 0 correlations. Formative indicators capture heterogenous sources and thus violate the fundamental assumption of factor analysis (a single latent cause is responsible for all items)

Concerning the ageism experiences in this study, we conceptualised the self-directed ageism items as reflective indicators. These items capture internalised beliefs about one’s age (e.g., “I am a burden because of my age”). In contrast, the interpersonal and institutional ageism items represent formative indicators. These items capture heterogeneous, external experiences across different domains (e.g., being excluded by others, unfair treatment, experiencing age-based barriers in some settings). A person may experience interpersonal exclusion but no institutional barriers. Thus, the overall construct of experienced interpersonal or institutional ageism is formed by the presence or absence of these diverse experiences, rather than being an expression of a single latent psychological trait.

Given the limited space in the discussion and the conciseness of the paper, we have added specific citations that explain the conceptual and statistical differences between formative and reflective indicators. This allows readers to understand the distinctions and reference the foundational literature. We also note that the interpretation of self-directed and interprofessional/institutional aspects must take into account the nature of these different indicators.

The revised texts are as follow: “Analogous considerations when applied to ageism indicators suggest that self-directed ageism could be conceptualised as a latent psychological trait that determines specific self-directed ageist cognitions, emotions, and behaviours, making these indicator-construct relations reflective. However, a formative interpretation is arguably more suitable for the interpersonal and institutional ageism items as ageism experiences from the external environment can combine from heterogeneous sources, namely, a diversity of individuals and institutions, rather than a single unitary factor. For more details on the conceptual and statistical distinction between formative and reflective indicators, please refer to (Bollen & Bauldry, 2011; Chang et al., 2016; Finn & Wang, 2014; Murray & Booth, 2018).”

**Comments1-12:** Positive vs. Negative Ageism: The decision to focus on negative ageism is unclear. The abrupt introduction of the positive vs. negative ageism in the discussion section, without previous reference or link to the measure's development is unclear. It's essential to justify how this decision informed the scale's content and design.

**Response:** We thank the reviewer for highlighting the ambiguity concerning the scale’s focus on ageism content and for noting that this rationale should be established earlier in the manuscript.

The WHO Ageism Experiences Scale was designed to exclusively measure negative ageism (i.e., harmful stereotypes, prejudice, and discrimination). This core design decision was made during the initial scale development phase (Murray & De La Fuente-Núñez, 2023). It was decided early on that since negative ageism is more consequential in terms of its harms, the scale would prioritise the negative ageism construct. Although there were minority of items (3 out of 15) were phrased positively (e.g., “at my age, my life has plenty of purpose”), This was purely a psychometric design choice to mitigate response biases (such as acquiescence bias) that occur in scales dominated by negative statements.

To address the reviewer's concern that this justification appeared abruptly, we have ensured this crucial distinction and rationale are explicitly stated in the Item Selection subsection of the Methods. The Discussion section has also been revised to remove the redundant and abrupt introduction of this topic, allowing the convergent validity findings to speak directly to the scale's negative ageism focus, showing that positive and negative ageism seems to be distinct theoretically and “Positive ageism is relatively under-researched and a valuable future direction for research would be to explore how positive ageism, alongside negative ageism, impacts individuals of different age groups (e.g., Levy et al., 2022).”

The following texts were added to the Method section:

“Crucially, the scale’s content was restricted to negative ageism (i.e., harmful stereotypes, prejudice, and discrimination). This decision was based on evidence showing negative ageism is associated with greater harm and adverse outcomes than positive (benevolent) ageism, thus prioritising the most consequential aspect of the construct. However, a mixture of positively and negatively worded items were selected to help manage the acceptability of the scale. This was based on feedback at earlier stages of expert content validity review where the risk of an overly negative scale was highlighted (Murray & De La Fuente-Nunez, 2023)”

The following texts were added to the Discussion section:

“In addition to the core psychometric findings of the present study, a further notable finding was the lack of association between positive and negative aspects of ageism. The finding that the WHO scale (which measures negative ageism) was strongly correlated with the PAQ negative ageism subscale but only weakly with the PAQ positive ageism subscale supports the convergent validity of our measure. This pattern also provides empirical support for the theoretical distinction between negative and positive ageism, suggesting they are distinct constructs.”

Editorial Errors:

**Comments1-13:** The word "was" should be deleted on page 12 "correlations was are provided in Table 3".

Response: We thank the reviewer for identifying this grammatical error. The word *was* has been deleted from the sentence on page 12 so that it now correctly reads: “The correlations between WHO-ageism experiences scores and PAQ ageism scores are provided in Table 3.”

Reviewer #2: In this paper, the authors describe the development of an experiences of ageism scale. In general, the development of the measure is well-conceived and thorough. There are several spots in the manuscript, however, where the authors could provide more information and improve clarity. Please see more detailed comments below.

**Comments2-1:** Abstract: did the authors test concurrent or convergent validity? They used both terms in the method and results section.

**Response:** We thank the reviewer for noting the inconsistent use of the terms “concurrent validity” and “convergent validity” across the manuscript. We clarify that convergent validity is the correct and intended term. As we noted, there is currently no “gold standard measure of ageism to compare the new WHO ageism experiences scale against”. To ensure consistency and accuracy, we have revised the Abstract and any other instances in the manuscript to exclusively use the term *convergent validity*.

**Comments2-2:** Introduction and method. On p. 4 and 5-7 the authors describe the process of developing an item pool and then winnowing it down to the 15 items tested for this paper. More detail should be provided about the larger overall item pool and how the item elimination process proceeded.

**Response:** We thank the reviewer for requesting more detail on the scale’s development, particularly concerning the initial item pool and the item selection/elimination process. We agree that providing this background strengthens the justification for the final 15 items tested in this preliminary validation study. Murray & De La Fuente-Nunez (2023) developed a comprehensive and conceptually grounded pool of ageism items aligned with contemporary multidimensional models. The item-generation process drew on extensive literature reviews and input from global ageism experts. This pool then underwent a rigorous content-validity review conducted by experts from all WHO-designated world regions, with expertise in ageism, psychometrics and older populations. This review led to refinements of the pool, including the removal of items lacking universal applicability across contexts and modification of others to enhance clarity and relevance.

The present study validated a 15-item subset of this broader pool. Specifically, the selection process was driven by a commitment to maximising content validity, prioritising both relevance and representativeness of the core ageism domains. Following the framework of(Haynes et al., 1995), we defined content validity as ‘the degree to which the elements of an assessment are relevant to and representative of the targeted construct for a particular assessment purpose’. Item selection was, therefore, driven by expert judgement regarding how well specific items satisfied these two criteria relative to others in the comprehensive item pool. Specifically:

- Cover all key domains align with Levy (2009)’s definition and the Global report on Ageism from WHO. Specifically, it spanned three components (stereotypes, prejudices, and discrimination), at three levels (self-directed interpersonal, and institutional level). To our knowledge, this is the first scale that can adequately capture all three aspects with respect to ageism experiences.
- Prioritise general items to capture overall exposure across contexts, making the scale feasible for use across diverse ages groups and countries.
- Balance the scale, including a mixture of positively and negatively worded items to manage the risk of an overly negative scale (which happens in many other scales about age discrimination, or age stereotypes)
- Capture a rage of severity range, from *everyday* to more *extreme*.
- Give proportionally greater representation to discrimination items to reflect the assumption that the behaviour aspect is the most direct and impactful form of ageism.

To further increase transparency regarding the selection process and content validity considerations, we have added a discussion of the specific criteria (relevance and representativeness) to the Method section of the manuscript. Additionally, we have included a brief discussion of items that were not selected (e.g., 'Others feel anger towards me because of my age' was excluded as it was deemed conceptually redundant or less optimal than a similar, less extreme item) and have indicated that the full non-selected item pool is available in the supplemental materials of the item development paper (Murray & De La Fuente-Núñez, 2023).

The following texts were added to the main texts: “The item selection process was primarily driven by expert judgement aimed at satisfying the criteria for content validity. Content validity was conceptualised as the extent to which the components of the measure appropriately and thoroughly reflect the target construct for a given assessment goal. Specifically, two key features of content validity are relevance and representativeness (Haynes et al., 1995). The former refers to the appropriateness of an aspect of a scale to the target construct and the latter to the extent to which the facets of the target construct are proportionally represented by items. Thus, for a scale to possess content validity, it should include items covering the full range of possible manifestations both in terms of specific types of symptoms and in terms of ‘severity’ in proportion to the target construct in the population.”

We have also revised the manuscript to provide clearer signposting and explicitly cite the development paper. The following texts were added:

“While a full account of the item generation and rigorous multi-stage elimination process is beyond the scope of this preliminary validation, further information is available in the development paper (Murray & De La Fuente-Nunez, 2023). It involved multiple stages of refinement. Items lacking broad contextual relevance were removed, and others were modified for clarity and precision. The current study presents the validation of a 15-item ageism experiences scale drawn from this finalised pool.”

**Comments2-3:** Sample. The authors collected their data using the platform Prolific. More demographic information should be provided about the sample, including the age range, education levels, income levels, countries of origin, self-rated health, etc.

**Response:** We thank the reviewer for highlighting the need for more detailed demographic information about the sample. We agree that this information is important for fully contextualising the findings. In the revised manuscript, we have now added the demographic variables that were collected (education, ethnicity, marital status) to the Methods (Participants) section. The age range was already reported in the original submission. The added texts read as follows: “89% of participants identified as White, with the remaining participants identifying as Asian/Asian British (4.2%), Black/African/Caribbean/Black British (2.0%), Mixed/Multiple ethnic groups (1.7%), or Others. Most respondents held a bachelor’s degree or higher, including 50.2% with a Bachelor’s degree, 18.4% with a Master’s degree or MBA, and 3% with a PhD or MD. In terms of relationship status, participants were primarily never married (50.2%) or married (41.3%).”

Regarding income, although we asked participants to report their monthly income, inspection of the data suggested that a notable proportion of respondents *may* have misinterpreted this as annual income. Because we cannot definitively verify or correct these inconsistencies, we decided not to include the income variable in the descriptive analyses to avoid introducing misleading results.

**Comments2-4:** Knowing the age range and also the breakdown of the number of young adult, middle-aged adult, and older adult participants is important, given the authors have the goal of developing a measure that can be used for multiple age groups. Given the mean age is 40, it appears that middle-aged adults (and likely younger adults) are overrepresented in the sample, and that older adults might be underrepresented.

**Response:** We thank the reviewer for raising this important point. We agree that a wider age group representation is essential for evaluating this preliminary validation, particularly given that the scale is intended to assess ageism across a broad age range. As noted, our sample has a mean age of 40.11 (SD = 11.01; Min = 20, Max = 65), suggesting that middle-aged and younger adults are overrepresented, while both adolescents and older adults are underrepresented.

Although the scale was designed to be applicable across the life course (i.e., from adolescents to older adults), this study represents an early validation in a mixed-age adult sample. We agree that this limits generalisability, particularly to older populations, and we now explicitly note this limitation in the manuscript. However, we believe that validating the scale in a middle-aged sample is an important step, given the absence of existing instruments that comprehensively capture ageism experiences across multiple age groups and thus limited studies on examining ageism among adults. We have also added citations to studies that have used and validated the scale among older adults, for readers interested in evidence from older populations.

The revised/added texts are as follows: “Crucially, while the sample was drawn from the UK, it was largely ethnically homogeneous (primarily White British), which restricts the generalisability of the findings across diverse ethnic subgroups. Similarly, though different age groups may have different experiences of ageism, we didn’t have sufficient numbers of participants to conduct age-stratified analyses, as well as an underrepresentation of older adults in this sample.” “However, further work will be required to extend the validation to other populations; including the adaptation and translation of the scale for use in a wider age range, a diversity of additional global contexts and ethnically diverse groups. Future validation studies in populations where ageism is expected to be more prevalent (e.g., older adult clinical samples or specific cultural setting) will be essential to test the scale’s full range sensitivity assess the extent of floor effect. It is also worth noting that the sample in this study comprised adults across a wide age range. Although several studies have validated this scale among older adults (e.g., Li, Bustamante, et al., 2025; Li, Sorocan, et al., 2025) and reported findings consistent with ours, generalisability to specific populations such as older adults should still be interpreted with caution.”

**Comments2-5:** It is clear that the authors consulted and put a lot of thought into the development of the item pool and eventual selection of the 15 items. They note that they made an effort to include some positively worded items, however, only 3 of the 15 are phrased positively. Isn’t it problematic that some of the types of ageism items they included were only captured using negative phrasing? Why weren’t at least 5 out of 15 items worded positively, so that a response set could be prevented as well as avoiding constructing an overly negative scale?

**Response:** We thank the reviewer for this thoughtful comment regarding the balance of positively versus negatively worded items, which is an important consideration in scale development. We agree that having a greater number of negatively phrased items can introduce the risk of response-set bias. However, the final balance reflects a necessary trade-off made during the item selection process. In developing this scale, we prioritised content validity over maintaining balance in item phrasing. Our primary aim was to ensure comprehensive coverage of all key levels and domains of ageism (self-directed, interpersonal, and institutional experiences, as well as stereotypes, prejudices, and discrimination). This need for broad content coverage was more important than keeping an even mix of positive and negative wording. Moreover, existing literature demonstrates that ageism is associated with many negative outcomes, and specifically, its negative components (e.g., negative beliefs, feelings, and experiences) are most strongly linked to adverse outcomes. Capturing these constructs inherently requires more negatively phrased items, as they more accurately reflect the lived experience of ageism. Thus, the leaning toward negative phrasing emerged naturally from the goal of capturing the construct comprehensively and validly. Finally, there were also some feasibility constraints. It was not always possible to generate suitable positive wordings for every item while maintaining clear, natural, and grammatically simple constructions. For several items, positively worded variants sounded strange or became overly complex, which would have negatively impacted the face validity and comprehensibility of the scale, thus potentially hindering data quality more than the slight imbalance in wording.

**Comments2-6:** The rationale the authors provide for why internal consistency is not calculated for all of the different subscales of the measure is confusing (p. 8). I have never encountered the arguments they are making for why some psychometric statistics are calculated for some items but not for others.

**Response:** We appreciate the reviewer raising this point. We acknowledge that the rationale for applying certain psychometric statistics (like Factor Analysis and Internal Consistency) selectively, i.e., calculating them for the self-directed ageism items but intentionally omitting them for the interpersonal and institutional ageism items, is complex and not commonly encountered in other psychometric practice. This decision is based on the conceptual distinction between reflective versus formative indicators, which we have detailed in Responses to comments 1-6, 1-7, and 1-11. To avoid repetition here, please see response to comments 1-6, 1-7, and 1-11 for more details. We have clarified this rationale in the manuscript to ensure that readers can easily understand the basis for this methodological choice.

**Comments2-7:** Did the authors collect education levels, income levels, employment status, and country of origin? It would be helpful to know if any of those sociodemographic factors correlated with the ageism measure, given health status does.

**Response:** We thank the reviewer for this comment. We have now performed additional analysis examining the association of education level and marital status with ageism scores. We have revised the Results section and Table 4 to include these new findings: education was found to be not significantly associated with either the self-directed or the interpersonal/institutional ageism. Marital status showed a significant association, with married individuals reported higher self-directed and interpersonal/institutional ageism compared with those who were not married. We hope these new analyses provide valuable preliminary evidence regarding the discriminant ability and interpretation of the scale scores across core sociodemographic variables.

**Comments2-8:** Discussion. The authors indicate this is a ‘mixed age UK sample’ but they do not specify that in their description of collecting data via the platform Prolific. They should be much clearer on where their sample was recruited from and the sociodemographic of the sample.

**Response:** We thank the reviewer for requesting greater clarity and detail regarding our sample recruitment and sociodemographics. We have now added more information regarding the sociodemographics of the sample, and explicitly stated that participants were sampled via the online platform Prolific.

**References**

Anthoine, E., Moret, L., Regnault, A., Sébille, V., & Hardouin, J.-B. (2014). Sample size used to validate a scale: A review of publications on newly-developed patient reported outcomes measures. *Health and Quality of Life Outcomes*, *12*(1), 2. https://doi.org/10.1186/s12955-014-0176-2

Barker, M., O’Hanlon, A., McGee, H. M., Hickey, A., & Conroy, R. M. (2007). Cross-sectional validation of the Aging Perceptions Questionnaire: A multidimensional instrument for assessing self-perceptions of aging. *BMC Geriatrics*, *7*(1), 9. https://doi.org/10.1186/1471-2318-7-9

Bollen, K. A., & Bauldry, S. (2011). Three Cs in measurement models: Causal indicators, composite indicators, and covariates. *Psychological Methods*, *16*(3), 265.

Chang, W., Franke, G. R., & Lee, N. (2016). Comparing reflective and formative measures: New insights from relevant simulations. *Journal of Business Research*, *69*(8), 3177–3185. https://doi.org/10.1016/j.jbusres.2015.12.006

Finn, A., & Wang, L. (2014). Formative vs. reflective measures: Facets of variation. *Journal of Business Research*, *67*(1), 2821–2826. https://doi.org/10.1016/j.jbusres.2012.08.001

Gunawan, J., Marzilli, C., & Aungsuroch, Y. (2021). Establishing appropriate sample size for developing and validating a questionnaire in nursing research. *Belitung Nursing Journal*, *7*(5), 356–360. https://doi.org/10.33546/bnj.1927

Haynes, S. N., Richard, D., & Kubany, E. S. (1995). Content validity in psychological assessment: A functional approach to concepts and methods. *Psychological Assessment*, *7*(3), 238.

Jackson, S. E., Hackett, R. A., & Steptoe, A. (2019). Associations between age discrimination and health and wellbeing: Cross-sectional and prospective analysis of the English Longitudinal Study of Ageing. *The Lancet Public Health*, *4*(4), e200–e208. https://doi.org/10.1016/S2468-2667(19)30035-0

Levy, B. (2009). Stereotype Embodiment: A Psychosocial Approach to Aging. *Current Directions in Psychological Science*, *18*(6), 332–336. https://doi.org/10.1111/j.1467-8721.2009.01662.x

Li, X., Bustamante, M., Tamayo, N., Castellanos, D., Jaramillo, Á., Brochero, G., Ramos, E., Herrera, E., Vargas, N., Escudero, J., Chahine, M., Timlin, A., Wright, A., & Murray, A. (2025). *Psychometric Evaluation of the WHO Ageism Experiences Scale Among Older Adults in Four Colombian Cities*. OSF. https://doi.org/10.31234/osf.io/azb6s_v1

Li, X., Sorocan, T., Ciubotaru, D., Malasevschi, D., Zatic, V., Constantinova, A., Chahine, M., Timlin, A., Wright, A., & Murray, A. (2025). *Psychometric Validation of the WHO Ageism Experiences Scale in Russian and Romanian*. OSF. https://doi.org/10.31234/osf.io/6nt49_v1

MacKenzie, S. B., Podsakoff, P. M., & Jarvis, C. B. (2005). The problem of measurement model misspecification in behavioral and organizational research and some recommended solutions. *Journal of Applied Psychology*, *90*(4), 710.

Mokkink, L. B., Terwee, C. B., Patrick, D. L., Alonso, J., Stratford, P. W., Knol, D. L., Bouter, L. M., & de Vet, H. C. (2010). The COSMIN study reached international consensus on taxonomy, terminology, and definitions of measurement properties for health-related patient-reported outcomes. *Journal of Clinical Epidemiology*, *63*(7), 737–745.

Murray, A. L., & Booth, T. (2018). Causal indicators in psychometrics. In *The Wiley handbook of Psychometric Testing* (Wiley-Blackwell, Vol. 1, pp. 933–951). Wiley Blackwell.

Murray, A. L., & de la Fuente-Núñez, V. (2023). Development of the item pool for the ‘WHO-ageism scale’: Conceptualisation, item generation and content validity assessment. *Age and Ageing*, *52*(Supplement_4), iv149–iv157. https://doi.org/10.1093/ageing/afad105

Murray, A. L., & De La Fuente-Nunez, V. (2023). Development of the item pool for the ‘WHO-ageism scale’: Conceptualisation, item generation, and content validity assessment. *Age and Ageing*.

Murray, A. L., & Li, X. (2025). Rapid review to inform the selection of a set of brief set of universal indicators for use in large-scale cross-national ageism research. *Aging & Mental Health*, *29*(4), 669–678. https://doi.org/10.1080/13607863.2025.2450265

Rippon, I., Zaninotto, P., & Steptoe, A. (2015). Greater Perceived Age Discrimination in England than the United States: Results from HRS and ELSA. *The Journals of Gerontology: Series B*, *70*(6), 925–933. https://doi.org/10.1093/geronb/gbv040
