# Supplementary material for: Preliminary validation of the 15-item WHO ageism experiences scale in a mixed-age UK sample
Source: PLoS One. 2026 May 5;21(5):e0347035. doi: 10.1371/journal.pone.0347035 (PMC13143091; doi:10.1371/journal.pone.0347035)
Supplement: Attachment — Submitted filename: Response to reviewer_ageism-15_R2_plosone.docx [file pone.0347035.s006.docx]

**PONE-D-25-50563**

**Response to Reviewers**

Dear Dr. Antony Bayer,

Thanks for giving us the opportunity to submit a revised draft of the manuscript “Preliminary validation of the 15-item WHO ageism experiences scale in a mixed-age UK sample” for publication in *PLOS One*. We appreciate the time and effort that you and the reviewers dedicated to providing feedback on our manuscript and are grateful for the insightful comments on and valuable improvements to our paper.

We have incorporated suggestions made by the reviewers. Those changes are highlighted within the manuscript. Please see below, in blue, for a point-by-point response to the reviewers’ comments and concerns, and quoted text from the manuscripts is in purple.

**Comments from Editor and reviewer:**

**1. The references need attention so that they are consistent and complete and in keeping with PLOS ONE style (Vancouver/NLM, including all authors, recognised journal title abbreviations, doi, etc.), numbered in text rather than author names and without duplicates (1 and 4).**

**Author Response:** We thank the reviewer for this observation. We have thoroughly revised the reference list to comply with PLOS ONE Vancouver/NLM style. Specifically:

- References 1 and 4 were identical (World Health Organization. Global report on ageism. 2021). We have removed the duplicate entry and renumbered all in-text citations accordingly.
- All references now include full author lists, recognised NLM journal title abbreviations, and DOIs where available.
- All in-text citations have been converted from author-date format to Vancouver numbered style throughout the manuscript.
- We have verified that all references cited in the text appear in the reference list and vice versa.

**2. Abbreviations should be spelt out when they first occur.**

**Author Response:** We have reviewed the full manuscript and ensured that all abbreviations are defined at first use. Abbreviations now introduced in full on first occurrence include: World Health Organization (WHO), exploratory factor analysis (EFA), parallel analysis with principal components (PA-PCA), minimum average partial test (MAP), socioeconomic status (SES), English Longitudinal Study of Ageing (ELSA), Health and Retirement Study (HRS), and Aging Perceptions Questionnaire (APQ).

**3. Also, on page 16 you refer to “older adults” and given that your upper age limit was 65, it would be helpful to clarify what age group is meant.**

**Author Response:** We thank the reviewer for highlighting this imprecision. We have revised the sentence to read: "…as well as an underrepresentation of participants in the older age range of the sample (i.e., those aged 50–65)." This more accurately reflects the composition of our sample, in which the maximum age was 65 and no participants above this threshold were included.
